# Treatment of severely open tibial fractures, non-unions, and fracture-related infections with a gentamicin-coated tibial nail—clinical outcomes including quality of life analysis and psychological ICD-10-based symptom rating

**DOI:** 10.1186/s13018-021-02411-8

**Published:** 2021-04-17

**Authors:** Nike Walter, Daniel Popp, Viola Freigang, Michael Nerlich, Volker Alt, Markus Rupp

**Affiliations:** 1 Department for Trauma Surgery, University Medical Center Regensburg, Franz-Josef-Strauß-Allee 11, 93053 Regensburg, Germany; 2 Department for Psychosomatic Medicine, University Medical Center Regensburg, Regensburg, Germany

**Keywords:** Antimicrobial-coated nails, Infection prevention, Quality of life

## Abstract

**Background:**

Implant-associated infections depict a major challenge in orthopedics and trauma surgery putting a high burden on the patients and health care systems, strongly requiring improvement of infection prevention and of clinical outcomes. One strategy includes the usage of antimicrobial-coated implants. We evaluated outcomes after surgical treatment using a gentamicin-coated nail on (i) treatment success in terms of bone consolidation, (ii) absence of infection, and (iii) patient-reported quality of life in a patient cohort with high risk of infection/reinfection and treatment failure.

**Methods:**

Thirteen patients with open tibia fractures (*n* = 4), non-unions (*n* = 2), and fracture-related infection (*n* = 7) treated with a gentamicin-coated intramedullary nail (ETN Protect^TM^) were retrospectively reviewed. Quality of life was evaluated with the EQ-5D, SF-36, and with an ICD-10-based symptom rating (ISR).

**Results:**

At a mean follow-up of 2.8 years, 11 of the 13 patients (84.6%) achieved bone consolidation without any additional surgical intervention, whereas two patients required a revision surgery due to infection and removal of the implant. No specific implant-related side effects were noted. Quality of life scores were significantly lower compared to a German age-matched reference population. The mean ISR scores revealed mild psychological symptom burden on the scale depression.

**Conclusion:**

The use of a gentamicin-coated intramedullary nail seems to be reasonable in open fractures and revision surgery for aseptic non-union or established fracture-related infection to avoid infection complications and to achieve bony union. Despite successful treatment of challenging cases with the gentamicin-treated implant, significantly reduced quality of life after treatment underlines the need of further efforts to improve surgical treatment strategies and psychological support.

## Introduction

Fracture healing can be a complex and tedious process. Diverse treatment strategies utilize fracture fixation devices to restore bone consolidation and enhance patients’ quality of life. However, implant-associated infections depict a major challenge in orthopedics and trauma surgery putting a high burden on the patients and health care systems [1]. The risk of developing a posttraumatic infection is multifactorial with reported rates of 1–2% for closed fractures ranging up to exceeding 30% for Gustilo-Anderson type III open tibia fractures [2–4]. The prevalence is expected to increase, as the incidence of long bone fractures is projected to rise, especially in the elderly [5]. Depending on injury severity, success rates only vary between 70-90% with a recurrence of the disease in 6–9% of the patients [6–8]. Consequences are often fatal. Several limitations such as immobility up to amputations of the affected limb, prolonged length of stay in hospital, multiple surgeries, side effects of antibiotic medication, and further socioeconomic issues are often not to be avoided despite a variety of prevention concepts [9]. Hence, the improvement of infection prevention in fracture care is strongly required [1]. With the ground-breaking discovery of the release of antibiotics from poly(methyl methacrylate) (PMMA) bone cement in 1970, the door to prevent colonization of implants and thusly biofilm formation was opened [10]. Nowadays different antimicrobial-coated implants are available [11]. However, their long-term durability is not guaranteed and high level of evidence studies which underpin their benefit are still lacking [12]. For use in trauma surgery, a gentamicin poly(D, L-lactide) (PDLLA) matrix coating for tibial nails has been developed and is available on the European market since August 2005 [1].

Clinical studies reporting on outcomes after gentamicin-coated nail treatment are rare with heterogeneous designs. Hence, we aimed at evaluating the outcome of surgical treatment with gentamicin-coated nails on (i) treatment success defined as bone consolidation as well as absence of recurrence of infection, and (ii) patient-reported quality of life in a patient cohort with the indications Gustilo-Anderson type III open tibia fractures, non-unions or fracture-related infections, as these depict challenging high risk of reinfection and treatment failure cases.

## Material and methods

Patients treated with a gentamicin-coated intramedullary nail (ETN PROtect^TM^) in our department between September 2012 and January 2020 were prospectively included. Informed consent was obtained from all individual participants included in the study. The study was approved by the institutional ethics committee of the University Clinic of Regensburg according to the Helsinki Convention (file number 20-1680-101). Patients’ characteristics were retrospectively retrieved from the hospitals electronic patient files system. Treatment indications included open tibia fractures, non-unions, and fracture-related infections (FRI). Open fractures were classified according to the Gustilo-Anderson classification [13]. Non-unions were considered as cessation of bone healing within 6 months after trauma and the expectation that no consolidation will be achieved without accurate treatment [14]. Infections were defined by the criteria of the FRI consensus definition [15]. Clinical records and radiographs were reviewed with a minimum follow-up time of 6 months after implantation of the gentamicin-coated nail. Achieved bone consolidation was determined with an evaluated RUST score > 10 [16]. Treatment failure was defined as required revision surgery due to FRI or non-union. Recurrence of infection with necessary revision surgery was assessed beginning with implantation of the intramedullary nails.

Patient-related outcome and quality of life was assessed using the German Short-Form 36 (SF-36) and EQ-5D scores as well as an ICD-10-based symptom rating (ISR) [17, 18]. The latter is an inventory frequently used in psychosomatic anamnesis. It consists of 29 items and covers various mental syndromes with subscales for depression, anxiety, obsessive/compulsive disorders, somatoform disorders, and eating disorders [19]. The EQ-5D is a well-established generic quality of life instrument developed by the EuroQol group comprising five questions concerning the functional domains mobility, self-care, everyday life activities, pain/discomfort, and anxiety/depression [20]. The items were converted into a single EQ index value using German norm data weights [21]. Additionally, the EQ-5D was evaluated using the visual analog scale (VAS) method [22]. The widely used SF-36 health survey captures the general health status with 36 questions in eight functional domains: physical function, role physical, bodily pain, general health, vitality, social function, role emotional, and mental health. Summary scores for the physical and mental component were calculated using normative data from a German national health interview and examination survey conducted in 1998 with 7124 participants [23]. Quality of life scores were compared to normative data [23, 24].

Data was analyzed using SPSS statistics version 24.0 (IBM, SPSS Inc., Armonk, NY). Descriptive statistics were calculated for all variables. Continuous variables were expressed as the mean and standard deviation. For comparisons between continuous variables independent t-tests were performed after determining the distribution was appropriate for parametric testing by Levene’s test. Significance was set at *p* < .001.

## Results

Thirteen patients (5 women; 8 men; mean age 43.9 ± 15.8 years) were included in the analysis (Table 1). The mean follow-up time was 2.8 years with a minimum follow-up time of 6 months after implantation of the gentamicin-coated nail. Four patients (30.77%) had a polytrauma.

**Table 1:** Patient characteristics

	Age (years)	Gender	Fracture type (Gustilo-Anderson)	Indication	Bone consolidation	Polytrauma	Treatment failure
1	22	Female	3a, open	Aseptic non-union	Yes	Yes	No
2	53	Male	3c, open	Fracture	Yes	No	No
3	41	Female	3c, open	Fracture	Yes	Yes	No
4	51	Male	3a, open	FRI	Yes	No	No
5	61	Male	2, open	Aseptic non-union	Yes	No	No
6	64	Male	3a, open	FRI	Yes	Yes	No
7	27	Male	3b, open	Fracture	Yes	Yes	No
8	31	Male	closed	FRI	Yes	No	No
9	19	Female	closed	FRI	Yes	No	No
10	32	Female	3b, open	FRI	Yes	No	No
11	41	Male	3b, open	Fracture	Yes	No	No
12	51	Male	3b, open	FRI	No	No	Yes
13	71	Female	closed	FRI	No	No	Yes

The indication for the treatment with an ETN PROtect^TM^ a fracture-related infection in seven cases (53.85%), aseptic non-union in two cases (15.39%), and open fractures in four cases (30.76%). Initial fractures were classified as Gustilo-Anderson type II in one case (7.69%), as type IIIa in three cases (23.08%), as type IIIb in four cases (30.77%), and as type IIIc in two cases (15.39%). Three patients (23.08%) had closed fractures with primary application of an external fixator before intramedullary nailing was performed. Eleven patients achieved bone consolidation within the time of the follow-up (84.62%), whereas an infection occurred after treatment with an ETN PROtect^TM^ in two cases (15.39%). Subsequently, these were treated with removal of the gentamicin-coated nail after 3.4 months and 5.1 months, respectively.

**Fig. 1:**
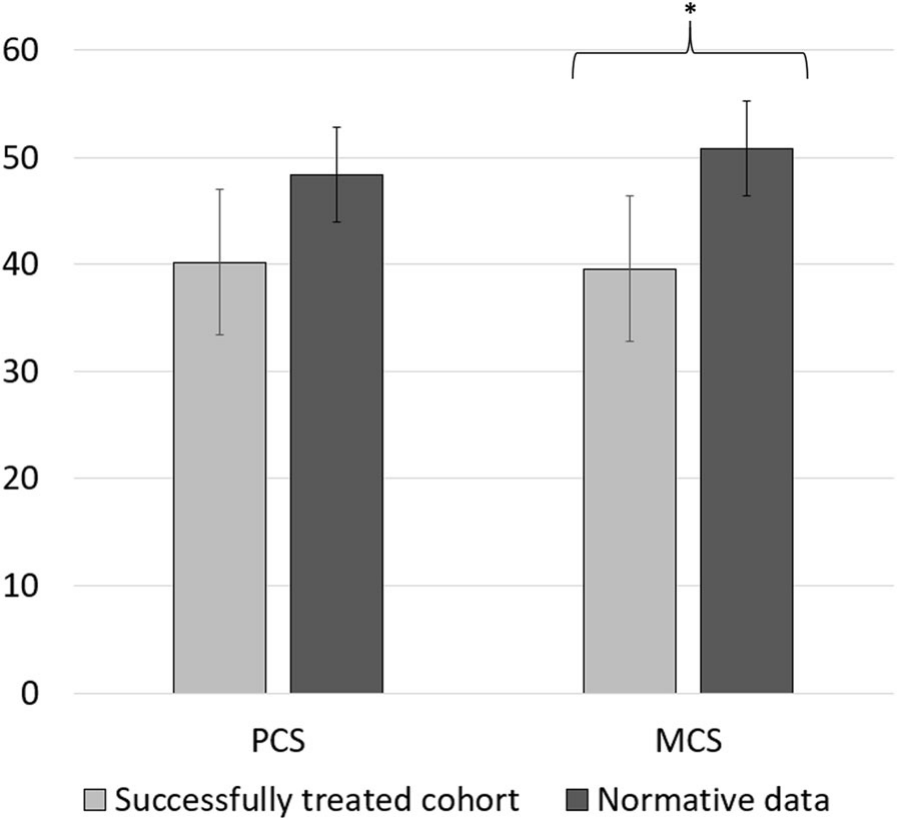
Mean physical health component score (PCS) and mean mental health component score (MCS) of successfully treated patients assessed with the SF-36.* Significant difference

**Fig. 2:**
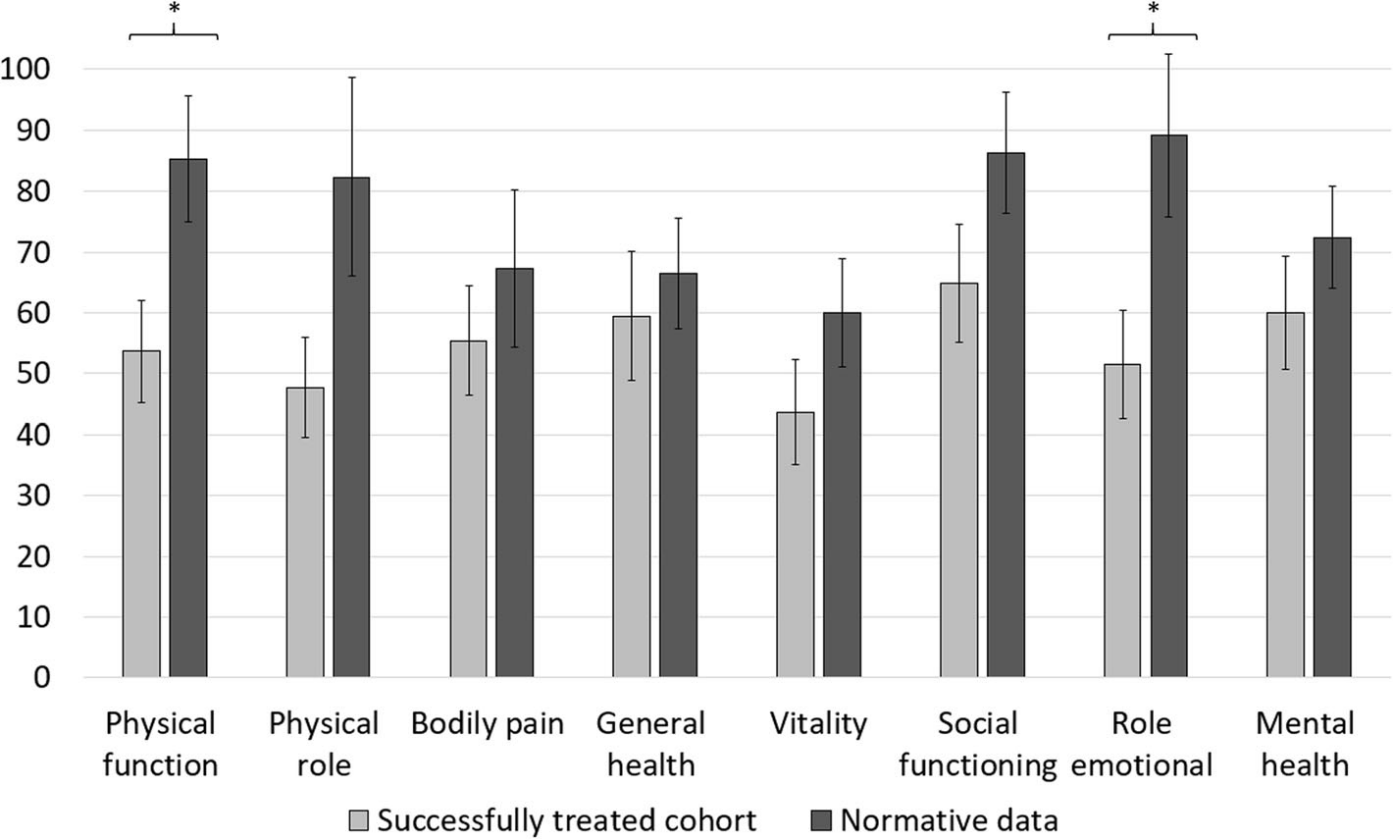
Subdimension scores for patient-related quality of life assessed with the SF-36.* Significant difference

Quality of life was evaluated for the eleven successfully treated patients (4 women; 7 men; mean age 40.8 ± 15.3 years) at a mean follow-up time of 3.2 years (range 0.8–7.7 years). The resulting mean physical health component score (PCS) of the SF-36 was 40.18 ± 13.1, and the mean mental health component score (MCS) of the SF-36 was 39.58 ± 13.6. In comparison with normative data from Germany, our patients score lower in the physical health component (PCS_Norm_ = 48.4 ± 9.2, *p* = .009) as well as in the mental health component of the SF-36 (MCS_Norm_ = 50.9 ± 8.8, *p* < .001), which depicts 83.1% and 77.8% of the summary scores obtained from the reference population, respectively (Fig. 1). The SF-36 subdomain analysis resulted in mean values of 53.6 ± 16.7 for physical function, 47.7 ± 16.4 for physical role, 55.5 ± 18.0 for bodily pain, 59.5 ± 21.3 for general health, 43.6 ± 17.2 for vitality, 64.7 ± 19.4 for social functioning, 51.5 ± 17.8 for emotional role, and 60.0 ± 18.8 for mental health. Hence, our cohort reached 62.8% for physical function (85.4 ± 20.7, *p <* .001), 58.0% for physical role (82.3 ± 32.7, *p* = .002), 82.3% for bodily pain (67.4 ± 25.9, *p =* .123), 89.5% for general health (66.4 ± 18.2, *p =* .192), 72.7% for vitality (60.0 ± 17.8, *p =* .006), 76.0% for social functioning (86.4 ± 19.9, *p =* .002), 57.8% for emotional role (89.1 ± 26.7, *p* < .001), and 82.8% for mental health (72.5 ± 16.7, *p =* .020) of the normative values (Fig. 2).

**Fig. 3:**
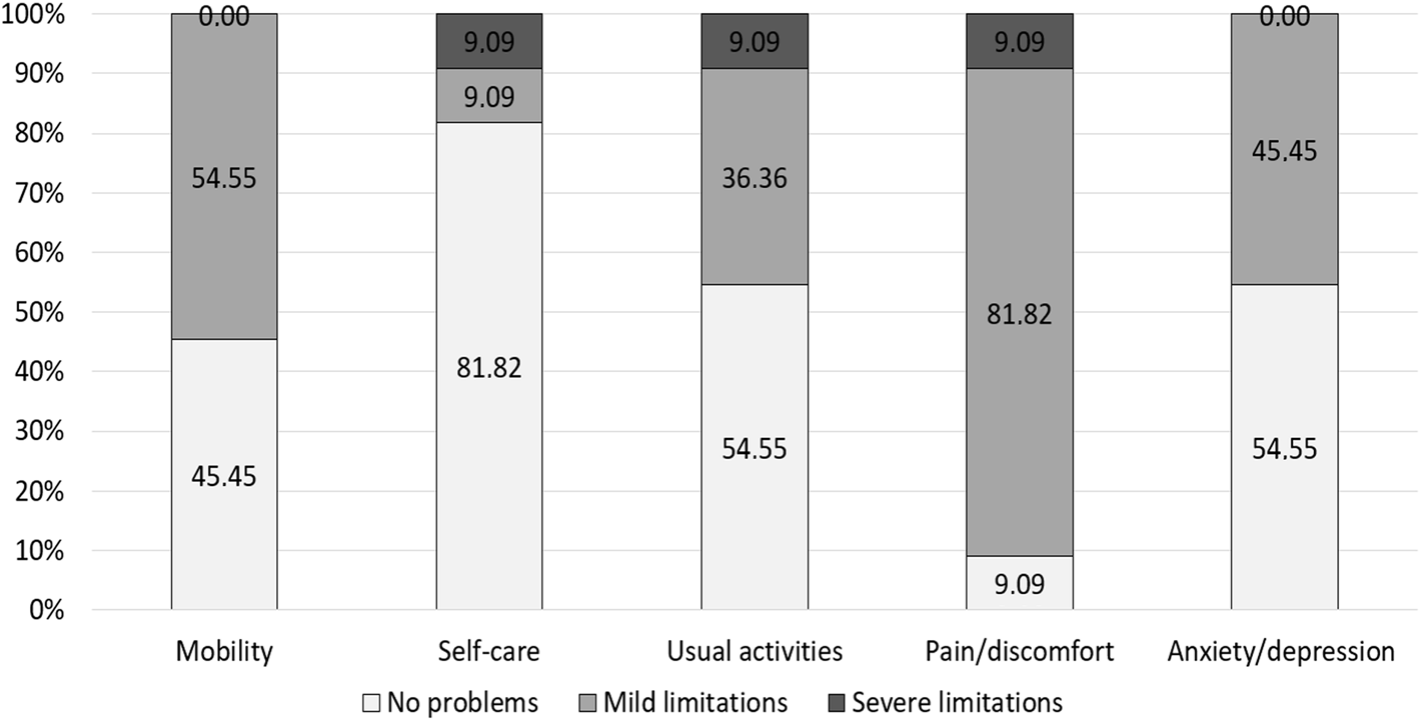
Results of the EQ-5D subdimensions given in percentage

The mean EQ-5D VAS rating reached 62.6 ± 24.5, which depicts 75.9% of the score of 82.5 ± 0.6 obtained from an age-matched reference population (*p* < .001). The mean EQ-5D index value was 0.769 ± 0.25, reaching 79.9% of the age-matched normative value 0.966 (*p* < .001) calculated based on a country-specific TTO value set [24]. In the subdimensions of the EQ-5D, patients showed limited results especially in mobility and pain/discomfort (Fig. 3). In total, 54.6% of the patients reported problems with mobility (compared to 15.9% of the German reference), 18.2% with self-care (compared to a norm value of 2.7%), 45.5% with usual activities (versus 9.9%), 90.9% with pain/discomfort (compared to 27.6%), and 45.5% with anxiety/depression (4.3% of the normative population).

**Fig. 4:**
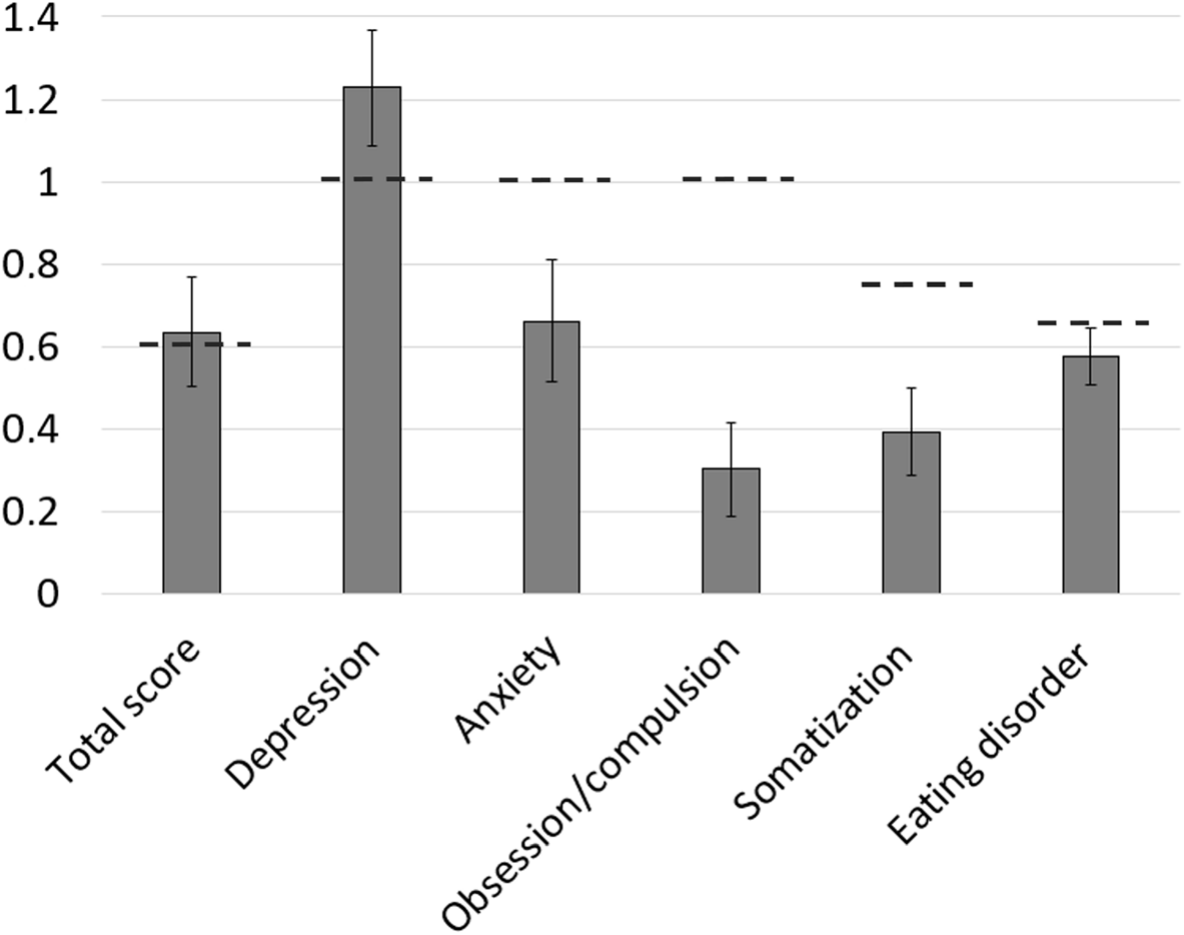
Mean values of the ISR scores obtained from successfully treated patients. The dotted line illustrates the border to considered mild psychological symptom burden

The mean total score of the ISR was 0.64 ± 0.26. The mean ISR subdimension scores reached 1.23 ± 0.28 for depression, 0.66 ± 0.30 for anxiety, 0.30 ± 0.23 for obsessive/compulsive disorders, 0.39 ± 0.21 for somatoform disorders, and 0.58 ± 0.14 for eating disorders, respectively (Fig. 4). Total scores above 0.6, scores above 1.0 for depression, anxiety, and obsessive/compulsive disorders, scores above 0.75 for somatoform disorders and scores above 0.67 for eating disorders are interpreted as mild psychological symptom burden [19].

The two cases considered as treatment failure (1 woman, 1 man; mean age 61.4 ± 13.7 years) reported a mean PCS of the SF-36 of 24.88 ± 4.12 and a mean MCS of 34.07 ± 4.6. The SF-36 subdomain analysis resulted in mean values of 12.5 ± 2.1 for physical function, zero for physical role, 37.0 ± 13.8 for bodily pain, 45.0 ± 4.5 for general health, 32.5 ± 2.2 for vitality, 56.3 ± 30.6 for social functioning, zero for emotional role, and 58.0 ± 10.5 for mental health. Lower scores in comparison to the successfully treated subgroup were also reflected in the mean EQ-5D index value of 0.633 ± 0.22 with a mean VAS rating of 45.0 ± 7.07. The mean total score of the ISR was 0.86 ± 0.43. The mean ISR subdimension scores reached 1.0 ± 0.82 for depression, 1.1 ± 0.48 for anxiety, 0.83 ± 0.29 for obsessive/compulsive disorders anxiety, 1.0 ± 0.0 for somatoform disorders, and 0.50 ± 0.50 for eating disorders.

## Discussion

In this study, the outcome of the use of a gentamicin-coated intramedullary tibia nail, ETN PROtect^TM^ was evaluated in a cohort of high risk of reinfection and treatment failure. Treatment success in terms of achieved bone consolidation and no required revision surgery due to infection was observed in eleven of thirteen patients.

Studies accessing the clinical outcome and especially patients’ quality of life after the use of gentamicin-coated nails in tibia fractures and revision cases are rare. Fuchs and colleagues evaluated 21 patients treated with a UTN PROtect Tibial Nail (DePuy Synthes, Bettlach, Switzerland) in a prospective case series with closed and open tibia fractures, as well as revisions, reporting no infection and radiographic union in 11 of 19 patients at a follow-up of 6 months [25]. Metsemakers et al. included nine patients with a Gustilo-Anderson grade II or grade III open tibial fracture, four infected non-unions, two acute tibial shaft fractures pretreated with external fixation, and one aseptic non-union with a soft-tissue defect in a retrospective analysis. The authors report no infection at a follow-up of 18 months [26]. Another analysis of 99 patients with open or closed tibial fractures as well as non-union revision cases treated with a gentamicin-coated titanium nail, resulted in observed infection in 4 of 55 patients after fracture and in 2 of 26 patients after revision surgery at a 18 months follow-up [27]. Vicenti and colleagues included 17 patients, who all achieved bone consolidation at a mean time of 7.18 months concluding that gentamicin-coated nails in association with the RIA system depict a safe and effective treatment of non-unions. They further showed a significant improvement of the quality of life, evaluated with the EQ-5D and the mean VAS for pain at a 3 months follow-up [28]. Additionally, better bone healing was reported at a 6 months follow-up in a cohort of 14 patients treated with gentamicin-coated nails compared to 14 cases treated with regular nailing and no gentamicin release into the systemic circulation above the lowest detectable level of 0.2 mg/dL could be determined in serum samples of patients treated with the ETN PROtect^TM^ [29, 30]. Hence, the application of a gentamicin-coated intramedullary nail (ETN Protect^TM^) seems reasonable to prevent implant-associated infections.

The patient-reported outcome measures reflect the high physical and mental burden resulting from the complex injuries even after successful bony consolidation and avoidance of infections/recurrence of infections. Quality of life of patients, which were successfully treated, was significantly lower compared to a German aged-matched reference population regarding the EQ-5D index value, the EQ-5D VAS rating, mental health component of the SF-36, as well as the SF-36 subdimensions physical function, physical role, and role emotional. The ISR showed mild psychological symptom burden in particular regarding depression. The two treatment failure cases showed reduced quality of life in comparison to successfully treated patients in the summary scores, as well as the subdimensions of the SF-36, the EQ-5D index value as well as the EQ-5D VAS rating and the mean total ISR score.

These finding are in line with other studies providing first insights into the serious consequences following fractures, non-unions, and fracture-related infections on patients’ physical and mental wellbeing [31–33]. For instance, Brinker et al. assessed the quality of life in 237 patients with tibial shaft fracture non-unions (14.8% infected) and report a mean SF-12 mental health component score of 42.3 ± 7.1 and a mean SF-12 physical health component score of 27.4 ± 6.7. Comparing their findings to different medical conditions, they show that the impact of a non-union situation regarding the physical, as well as mental health component is worse than congestive heart failure, type-2 diabetes mellitus, and myocardial infarction among others [32]. Recently, a meta-analysis was calculated for a direct comparison between the non-union and union situation showing a mean difference of − 11.94 scores for the SF-12 physical health component and a difference of − 6.42 for the SF-12 mental health component [31]. These results are in accordance with our findings, showing a difference of − 15.3 for the PCS and − 4.9 for the MCS assessed with the SF-36 between successfully treated patients who achieved bone consolidation and cases considered as treatment failure. Further, the impact of required revision surgeries has been highlighted by a study comparing outcomes after femoral shaft fractures treated with plate fixation and intramedullary nailing. Here, patients who did not require a reoperation achieved an EQ-5D index value of 0.95 after 1 year in comparison to a value of 0.83 for revision cases [34]. Additionally, the reported EQ-5D index value of 0.769 ± 0.25 is comparable to patients with multiple trauma, for which an value of 0.76 ± 0.27 has been calculated 24 months postoperatively [35] as well as to patients requiring trauma related surgery, who achieved an index value of 0.73 ± 0.29 with a follow-up time of 12 months [35, 36]. Also, the EQ-5D VAS score was similar to values reported from patients with pelvic ring fractures after a mean follow-up of 4.2 years [37]. Therefore, the reduced quality of life might be rather attributable to the severity of injuries than to the use of the ETN nail for the treatment. In general, patient-related outcome measures and the explicit investigation of the psychological impact of injuries should be deemed as a future direction of research as an underestimation of the burden of injury may affect resource allocation, necessary prevention priorities and may hinder the implementation of counseling as part of the standard care in trauma surgery. Newly emerging treatment strategies and prevention methods as well as interdisciplinary approaches should be implemented to restore and improve the overall quality of life of patients with complicated fractures, non-unions, and fracture-related infections.

Limitations of our study are the usual suspects. The study is a retrospective case series including patients with different indications for intramedullary nailing with a gentamicin-coated intramedullary nail (ETN PROtect^TM^). Due to the small sample size, subgroup analysis was not deemed applicable as results may be statistically underpowered. Whereas the case number is comparatively low, the period in which patients have been treated with the ETN nail is long. Reasons for less numbers are the use of gentamicin-coated intramedullary nails only in patients with high risk of bone infection and long-term follow-up for clinical and radiological outcome. Treatment standards in fracture care did not change during this time frame. Thus, we believe the long study period rather enables a sound follow-up period than limit the value of the reported data.

## Conclusion

In conclusion, the application of a gentamicin-coated intramedullary nail (ETN PROtect^TM^) seems to be reasonable to avoid implant related bone infection in complex fractures and revision cases, when respecting current treatment standards in fracture care and infection surgery. Low quality of life after treatment underlines the need of further efforts to improve surgical treatment strategies and psychological support in complicated and challenging cases.

## Data Availability

The datasets analyzed during the current study are available from the corresponding author on reasonable request.

## Abbreviations

FRI: Fracture-related infection
ISR: ICD-10-based symptom rating
PMMA: Poly(methyl methacrylate)
PDLLA: Poly(D, L-lactide)
